# Dendrite-Free and Stable Lithium Metal Battery Achieved by a Model of Stepwise Lithium Deposition and Stripping

**DOI:** 10.1007/s40820-021-00687-3

**Published:** 2021-08-09

**Authors:** Tiancun Liu, Jinlong Wang, Yi Xu, Yifan Zhang, Yong Wang

**Affiliations:** 1 Department of Chemical Engineering, School of Environmental and Chemical Engineering, Shanghai University, 99 Shangda Road, Shanghai, 200444 People’s Republic of China; 2 Key Laboratory of Organic Compound Pollution Control Engineering (MOE), 99 Shangda Road, Shanghai, 200444 People’s Republic of China

**Keywords:** Lithiophilic skeleton, Stepwise Li deposition and stripping, Dendrite suppression, Lithium metal battery, Electrochemical properties

## Abstract

**Highlights:**

- A facile method is adopted to obtain cucumber-like lithiophilic composite skeleton.
- Massive lithiophilic sites in cucumber-like lithiophilic composite skeleton can promote and guide uniform Li depositions.
- A unique model of stepwise Li deposition and stripping is determined.

**Abstract:**

The uncontrolled formation of lithium (Li) dendrites and the unnecessary consumption of electrolyte during the Li plating/stripping process have been major obstacles in developing safe and stable Li metal batteries. Herein, we report a cucumber-like lithiophilic composite skeleton (CLCS) fabricated through a facile oxidation-immersion-reduction method. The stepwise Li deposition and stripping, determined using in situ Raman spectra during the galvanostatic Li charging/discharging process, promote the formation of a dendrite-free Li metal anode. Furthermore, numerous pyridinic N, pyrrolic N, and Cu_x_N sites with excellent lithiophilicity work synergistically to distribute Li ions and suppress the formation of Li dendrites. Owing to these advantages, cells based on CLCS exhibit a high Coulombic efficiency of 97.3% for 700 cycles and an improved lifespan of 2000 h for symmetric cells. The full cells assembled with LiFePO_4_ (LFP), SeS_2_ cathodes and CLCS@Li anodes demonstrate high capacities of 110.1 mAh g^−1^ after 600 cycles at 0.2 A g^−1^ in CLCS@Li|LFP and 491.8 mAh g^−1^ after 500 cycles at 1 A g^−1^ in CLCS@Li|SeS_2_. The unique design of CLCS may accelerate the application of Li metal anodes in commercial Li metal batteries.
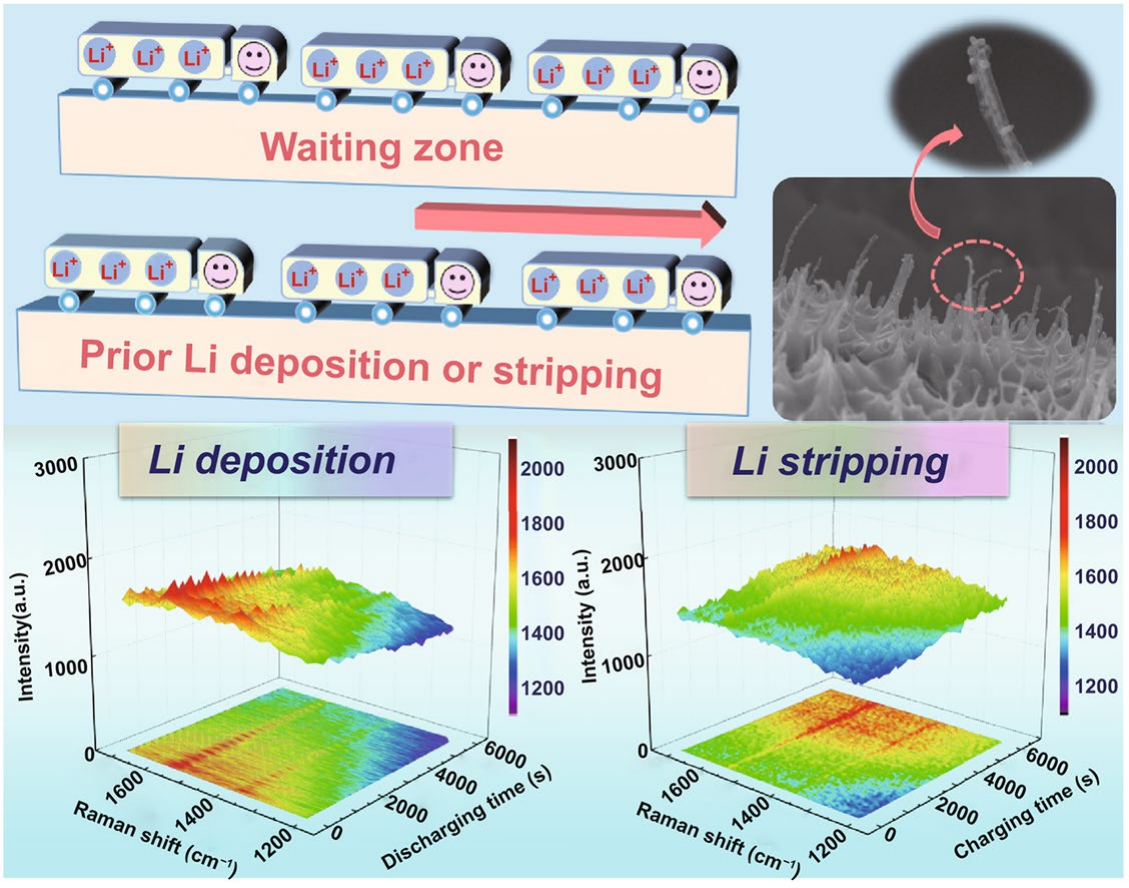

**Supplementary Information:**

The online version contains supplementary material available at 10.1007/s40820-021-00687-3.

## Introduction

Lithium (Li) metal anodes exhibit a high theoretical capacity (3860 mAh g^−1^) and the lowest reduction potential (− 3.04 V vs. SHE) among next-generation high-energy systems to enhance the Li-ion battery [1–3]. However, issues such as the fatal growth of dendritic Li, inhomogeneous Li deposition, and additional electrolyte consumption during the Li plating/stripping process result in low Coulombic efficiency (CE), quick capacity decay, and the internal short circuit of the cell. These critical challenges need to be overcome effectively for the practical utilization of Li metal anodes [4–6]. The primary concern is restraining the production and growth of Li dendrites. Recent literature has yielded several effective methods such as constructing an artificial solid electrolyte interface (SEI) with high stiffness [7–10], designing functional separators [11–13], and modifying the electrolyte by adding additives [14–16] to suppress the growth of Li dendrites. Consequently, these solutions have improved the cycling stability and prolonged the lifespan of the Li metal anode.

Constructing functional current collectors is another effective approach to achieve dendrite-free Li metal anodes. The Chazalviel diffusion model suggests that decreasing the effective electrode current density extends the Sand’s time, i.e., initial time for the growth of Li dendrites [17–19]. Consequently, the increase in the specific surface area reduces the local current density of the electrode while further suppressing the generation of Li dendrites. Several substrates with high specific surface areas have been designed as containers for the deposited metallic Li to produce dendrite-free Li metal anodes [20–24]. Additionally, lithiophilic sites in the substrate effectively disperse Li ions, thereby reducing the Li nucleation barrier and guiding uniform Li deposition. Some inorganic components such as heteroatom-doped carbon [25–27], metals and their derivatives [28–35] along with unique organic materials [36–39] possess excellent lithiophilicity to promote homogeneous Li deposition and growth. Therefore, an ideal current collector must possess abundant lithiophilic groups to impart substantial lithiophilicity.

Herein, we propose a distinct cucumber-like lithiophilic composite skeleton (CLCS) as a loader of deposited Li metal to enhance the electrochemical performance and improve the lifespan of Li metal batteries. Specifically, numerous lithiophilic groups (pyridinic N, pyrrolic N, and Cu_x_N sites) in the CLCS exhibit strong binding to Li atoms, resulting in improved homogeneous dispersion of Li ions and subsequent dendrite-free Li deposition. Furthermore, stepwise Li deposition and stripping can be conducted during the galvanostatic Li charging/discharging process, suppressing concentrated Li nucleation while avoiding aggressive Li growth. Consequently, both asymmetric and symmetric cells based on CLCS exhibit a prolonged cycling lifetime and a decreased voltage polarization. Furthermore, full cells composed of CLCS@Li anodes deliver a stable cycling plot and high capacity retention when coupled with common cathode materials (LiFePO_4_ and SeS_2_).

## Experimental Section

### Materials

Commercial copper foam (CF) substrate with the thickness of 0.5 mm was provided by KunShan GuangJiaYuan new materials Co., Ltd. N, N-dimethylformamide (DMF) solvent and poly(acrylonitrile) (Mw = 150,000) were obtained from J&K Scientific and Sinopharm Chemical Reagent Co., Ltd..

### Material Synthesis

Commercial CF substrates were cleaned and placed in a muffle furnace and annealed at 600 °C for 2 h in the presence of air. Subsequently, the black oxidized CF substrates were immersed in a pre-prepared poly(acrylonitrile) solution (8 wt%) for 10 min and then transferred to an oven for drying. The CLCS was prepared by annealing the dried mixture at 600 °C for 2 h in a H_2_/Ar (5/95, v/v) atmosphere. The heating rate was set at 2 °C min^−1^.

### Material Characterization

The morphologies of various samples were observed and analyzed using a scanning electron microscopy (Hitachi SU1510) and transmission electron microscopy (JEM-2010F). The components of the CLCS were characterized using X-ray diffraction (XRD, Rigaku D/max-2550 V). Raman spectra and in situ Raman spectra were obtained using a Raman spectrometer (Renishaw inVia plus). Related in situ optical images were conducted by Smartroom 5 (Zeiss). The XPS measurements were conducted on a Thermo Scientific K-Alpha instrument.

### Electrochemical Measurements

Each coin cell (CR 2032) based on CF or CLCS electrodes was assembled in an argon-filled glove box with H_2_O and O_2_ content lower than 0.1 ppm. The Coulombic efficiency was determined using a specific amount of Li metal plated on the bare CF and CLCS, which was then stripped away through the voltage of 1 V. For galvanostatic charge/discharge testing, CF@Li or CLCS@Li electrodes loaded with a Li deposition of 3 mAh cm^−2^ were assembled into symmetrical cells. Specially, the specific capacity of the CLCS@Li composite anode in symmetry and full cells is ~ 1600 mAh g^−1^. Celgard 2400 and 1 M LiTFSI in DOL/DME (1:1, v/v) with 2% LiNO_3_ additive were chosen as the separator and electrolyte, respectively, in asymmetrical and symmetrical cells. The amount of selected electrolyte was controlled at 40 uL per coin cell. The full cells assembled using commercial LiFePO_4_ (LFP) and selenium sulfide (SeS_2_) cathodes. The SeS_2_ cathodes were fabricated by preparing a mixture of weighed cathode powder, conductive carbon black, and polyvinylidene fluoride (PVDF) binder in a mass ratio of 8:1:1 dispersed in N-methyl-2-pyrrolidone (NMP) solvent and casting it on Al foil. Subsequently, the Al foil with slurry was dried in a vacuum oven at 120 °C for 10 h. The dried foil loaded with an LFP (areal mass: ~ 6 mg cm^−2^) or SeS_2_ (areal mass: ~ 1.5 mg cm^−2^) cathode was assembled into a full cell with a specific Li metal anode. 1 M LiPF_6_ in EC/DEC (1:1, v/v) and 1 M LiTFSI in DOL/DME (1:1, v/v) with 2% LiNO_3_ additive were used as the electrolytes of the full cells composed of LFP and SeS_2_, respectively. Furthermore, the amount of electrolyte used was fixed at 40 µL per coin cell. The voltage window of 2.5–4.2 V was applied to the Li|LFP cell. Similarly, the Li|SeS_2_ full cell was controlled between 1.7 and 2.8 V. The electrochemical performance was measured in a LAND-CT2001 test system.

### Theoretical Calculation

The constructed model of CLCS composite was optimized by the DMol^3^ module of Materials Studio (MS) software, in which the functional was chosen as Perdew–Wang (PWC) in local density approximation (LDA).

## Results and Discussion

### Preparation and Characterizations of CLCS

**Fig. 1:**
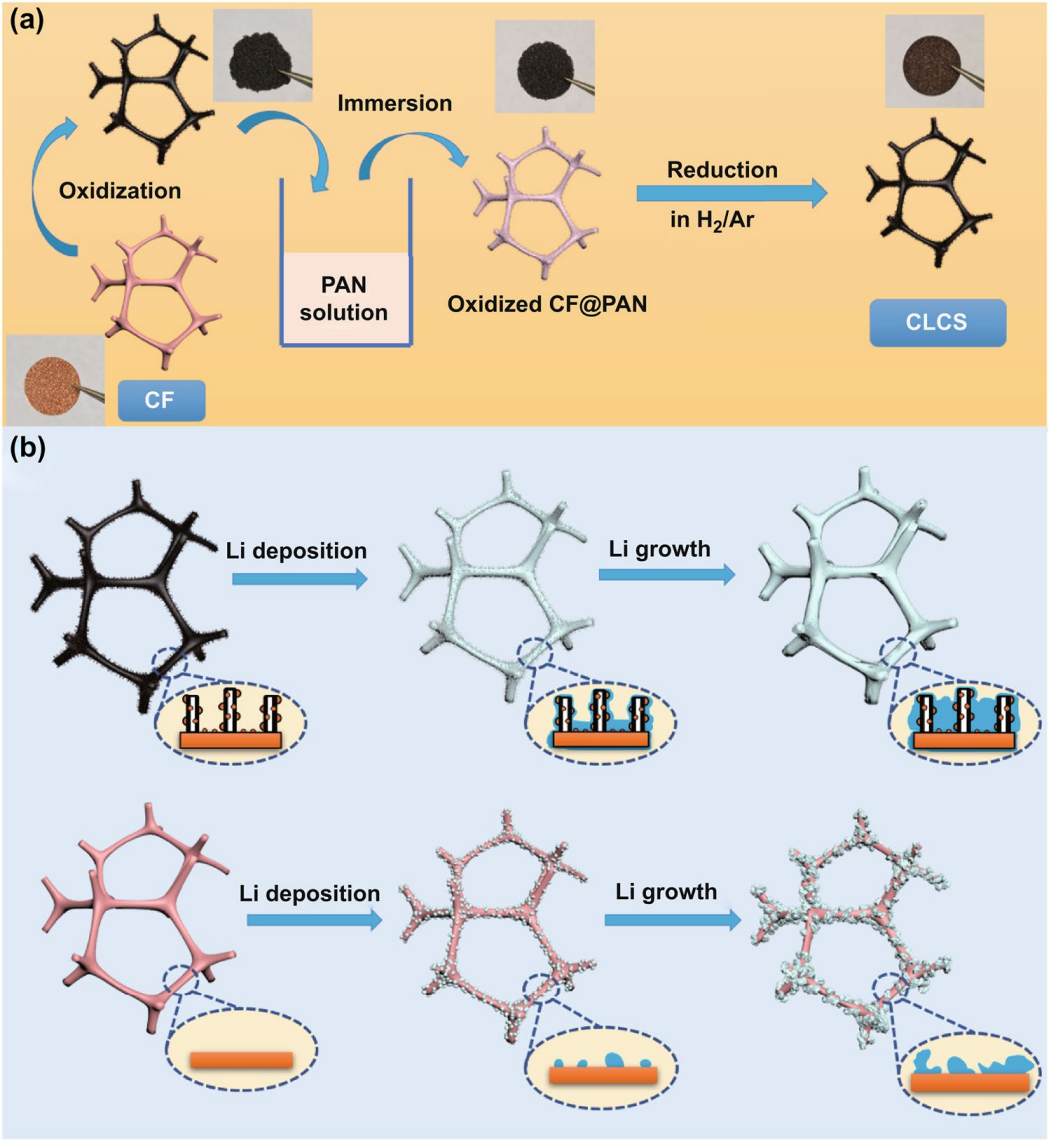
**a** Schematic diagrams for the synthetic process of CLCS. **b** Different Li deposition and growth behaviors in bare CF and CLCS

A facile oxidization-immersion-reduction method was explored to produce a novel functional current collector. Figure 1a illustrates the preparation of the CLCS in detail. Initially, metallic copper foam (CF) was oxidized in air and transformed into the fragile and black copper oxide (CuO) skeleton. Subsequently, the free-standing CuO skeleton was immersed in an as-prepared commercial-grade polyacrylonitrile (PAN) solution for organic coating. Finally, CLCS was obtained through a reduction process at 600 °C in a H_2_ and Ar (5%/95%, v/v) atmosphere from a CuO composite skeleton coated with PAN with the color of the products changing from black to brown. As illustrated in Fig. 1b, the different morphology observed following Li deposition on bare CF and CLCS highlights the difference in the lithiophilicity between these two substrates. Furthermore, uniform Li nucleation and growth can be achieved in the CLCS where Li ions are attracted to lithiophilic N-containing groups (pyridinic N, pyrrolic N, and Cu_x_N sites) during the Li plating process and then reduced on the CLCS. These excellent lithiophilic sites can suppress dendritic Li growth from the CLCS, and the resultant uniform Li deposition can enhance working safety, improve the electrochemical performance, and prolong the cycling life of cells. Conversely, an inhomogeneous distribution of metallic Li grains occurs in the CF substrate owing to its poor lithiophilicity at the initial Li nucleation stage. The continuous Li deposition would transform the plated Li metal into large bulks with various forms such as Li dendrites, resulting in unnecessary electrolyte consumption and the internal short circuit of the working battery.

**Fig. 2:**
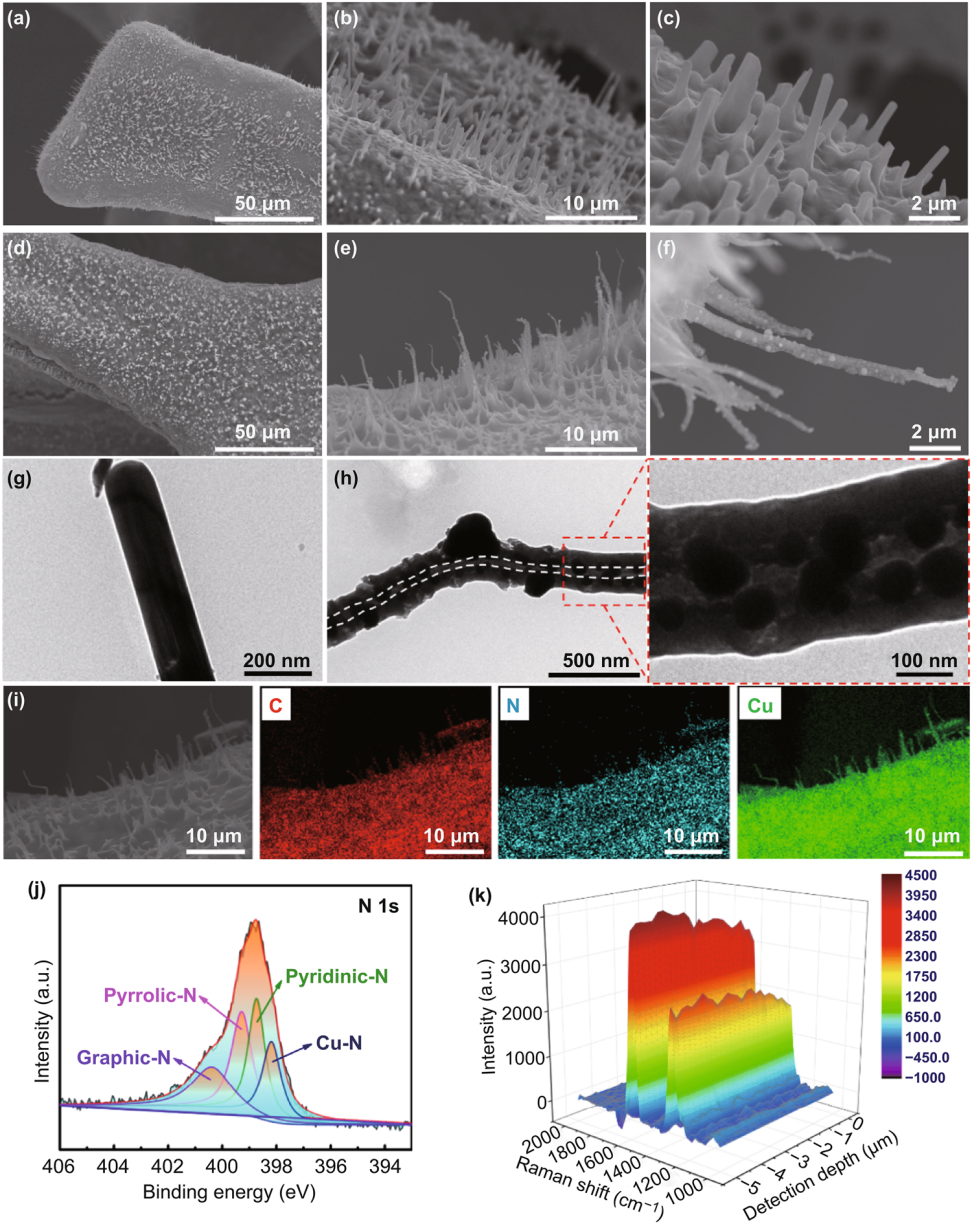
SEM images of **a-c** oxidized CF and **d-f** CLCS. TEM images of **g** oxidized CF and **h** CLCS. **i** EDS mapping containing C, N and Cu element for CLCS. **j** XPS survey spectra (N 1s) and **k** Raman spectra at different depths of CLCS

As displayed in Fig. S1, the bare CF exhibited a shiny color and a smooth surface. After calcination in air, the oxidized CF turned black owing to the formation of CuO (Fig. 1a). Figure 2a–c displays the related scanning electron microscopy (SEM) images of the oxidized CF which anchors several vertical CuO nanoneedles with flat surfaces. However, these unique nanoneedles could not maintain their structure during the calcination process in a mixed gas atmosphere (H_2_/Ar: 5%/95%, v/v). The reduced CF following the calcination process illustrated in Fig. S2 indicates the absence of the original nanoneedles and pores in the skeleton. Conversely, massive nanoneedles were retained in the CLCS with the perfect protection of the coated carbon layer. As shown in Fig. 2d–f, several thin nanoneedles are decorated with Cu nanoparticles in the CLCS. The SEM image at higher magnification (Fig. S3) illustrates the surface of the CLCS being covered with massive N-doped carbon-coated Cu nanoparticles, which promotes uniform Li deposition. Figure 2g displays the transmission electron microscopy (TEM) image of a pristine CuO nanoneedle with a smooth surface and a solid structure, in agreement with Fig. 2c. However, as shown in Fig. 2h, a noticeable hollow space was observed in a nanoneedle of the CLCS, which could improve the electrolyte transport. Furthermore, there are some internal regular Cu nanoparticles formed from the decomposition and reduction of pristine CuO nanoneedles in addition to the external nanoparticles embedded in the nanoneedles. The elemental distributions of C, N, and Cu displayed in Fig. 2i are detected accurately and observed in the vertical nanoneedles using energy-dispersive X-ray spectroscopy, illustrating the structural uniformity of the CLCS. Furthermore, the distinctive distributions of the aforementioned elements are in accordance with the selected detection area in the SEM image (Fig. S4). This result substantiates the uniform distribution of C, N, and Cu elements in the CLCS. Figure S5 illustrates the XRD patterns of the CLCS. The peak at 23° was assigned to the amorphous carbon, and three typical characteristic peaks belonging to Cu metal (JCPDS No. 04-0836) were identified. As depicted in Fig. 2j, the XPS results were used to validate the structural composition of the CLCS. The common graphic N (399.31 eV), pyrrolic N (399.31 eV), pyridinic N (398.75 eV), and notably the Cu–N site (398.21 eV) are observed in the N 1s spectrum of the CLCS [40–43]. The isolated Raman spectrum of the CLCS displays a high degree of graphitization (Fig. S6). Additionally, the Raman spectra at different depths of the CLCS shown in Fig. 2k illustrate the slow gradual increase and eventual stabilization of the intensity of the D-band (1320.71 cm^−1^) and G-band (1579.97 cm^−1^) indicating interactions between the N-doped carbon and the reduced Cu nanoparticles, which affect the structural uniformity.

### Li Deposition and Stripping in CLCS

**Fig. 3:**
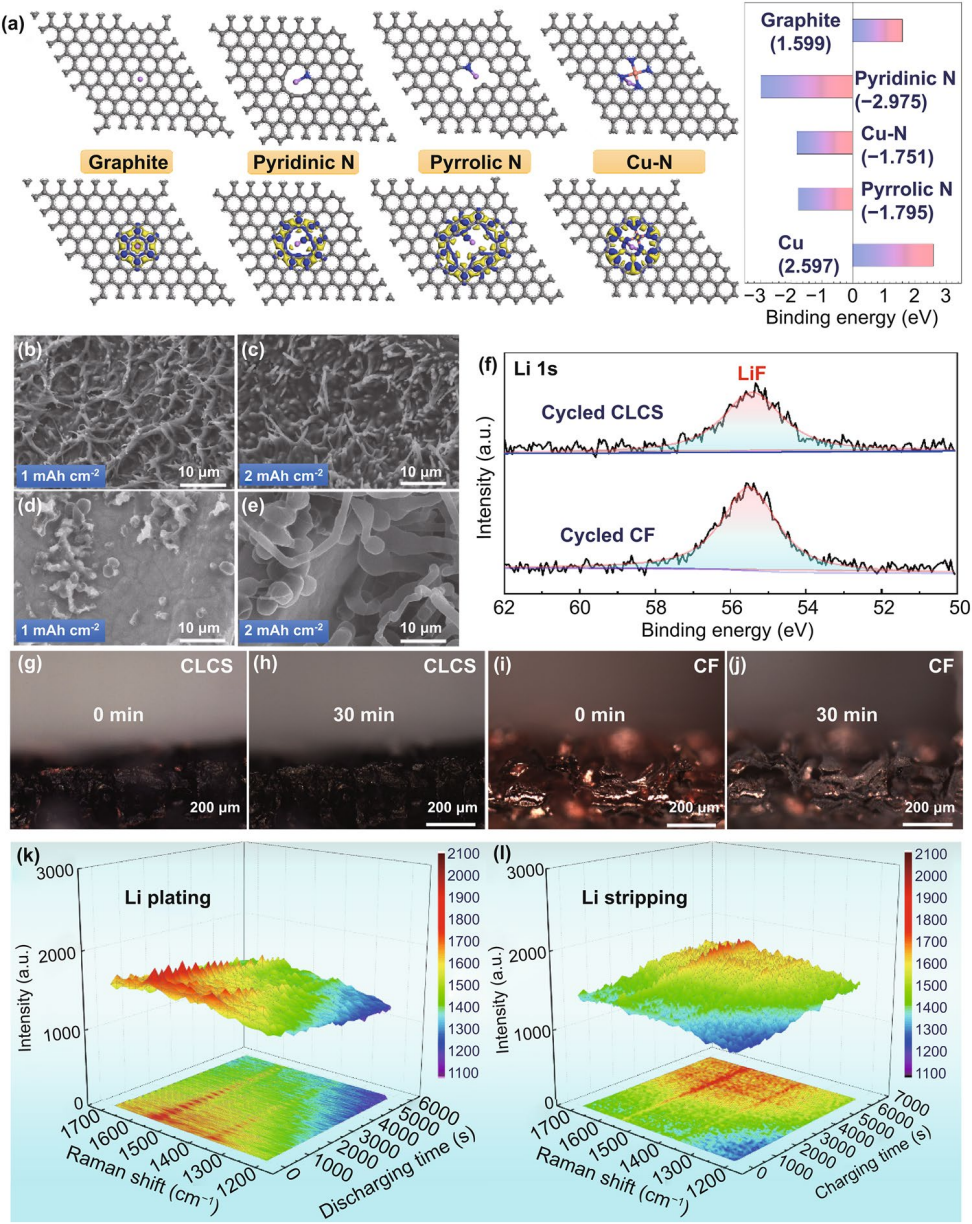
**a** Density functional theory calculations on the adsorption energy between Li atoms and graphite, pyridinic N, pyrrolic N and Cu–N site. Moreover, values of related adsorption energy for these groups are calculated. SEM images of CLCS loading with Li deposition of **b** 1 mAh cm^−2^ and **c** 2 mAh cm^−2^. SEM images of CF plating with metallic Li of **d** 1 mAh cm^−2^ and **e** 2 mAh cm^−2^. **f** XPS survey spectra of CLCS and CF electrode after 50 cycles. In situ optical observation of CLCS at the discharging time of **g** 0 min and **h** 30 min. In situ optical images of CF at the discharging time of **i** 0 min and **j** 30 min. In situ Raman spectra of CLCS at **k** Li plating and **l** Li stripping process

The remarkable lithiophilicity of the CLCS was explored using density functional theory (DFT) to calculate the binding energy and charge density of Li atoms adsorbed on preferential functional groups (Cu, graphite, pyridinic N, pyrrolic N, and Cu_x_N sites). As shown in Figs. 3a and S7, the binding energies of Li atoms and graphite and Li atoms and Cu were calculated to be 1.599 and 2.597 eV, respectively. This demonstrates the poor adsorption of Li atoms and the increased possibility of Li deposition and accumulation. However, the binding energy of the Li atom to pyridinic N (− 2.976 eV), pyrrolic N (− 1.795 eV), and Cu–N sites (− 1.751 eV) indicates a strong Li adsorption and effective Li distribution. Furthermore, the difference in the charge densities of the adsorbed Li on these aforementioned groups helps in determining their different lithiophilicity. The electron accumulation and reduction are represented by the blue and yellow regions, respectively. A lower charge transfer was observed for Li adsorbed on Cu and graphite, demonstrating the weak bond between Li and Cu as well as Li and graphite. Conversely, a significant degree of charge transfer was observed for the Li adsorbed on pyridinic N, pyrrolic N, and Cu–N sites. This further corroborates the strong bond formed between Li and N-containing functional groups while highlighting the impressive lithiophilicity of the CLCS. SEM images of the CLCS and CF loaded with different areal capacities of metallic Li are presented to evaluate the effect of guiding uniform Li deposition and suppressing the generation of Li dendrites. The surface of the CLCS without any dendrites was found to be smoother in comparison with the original morphology as Li was plated on the CLCS (Fig. 3b, c). These magnified images of the CLCS loaded with metallic Li indicate that the cucumber-like surface of the CLCS gradually disappears as the Li deposition capacity increases from 1 to 3 mAh cm^−2^, eventually flattening (Fig. S8a-c). In the process of Li stripping, the continuous stripping of Li metal from the CLCS causes the reappearance of a cucumber-like surface, indicating ordered, stepwise Li deposition and stripping (Fig. S8d–f). However, the Li deposition on CF exhibits a highly disordered distribution and an uncontrolled formation of dendritic Li due to the poor lithiophilicity of CF (Fig. 3d, e). Notably, irregular Li bulks and pronounced Li dendrites were observed from magnified partial details in the SEM images (Fig. S9). Additionally, the morphology of cycled CLCS and CF after 50 cycles indicated residual Li on the CF skeleton as compared to the residual-free CLCS (Fig. S10). Figure 3f displays the XPS spectra of the cycled CLCS and CF to assess the surface components. As illustrated by the Li 1 s spectra, LiF (55.48 eV) was detected in the cycled CF and CLCS, which can be ascribed to the decomposition of the electrolyte on the surface of the electrodes [44]. Furthermore, once the Li dendrites break the SEI, excess electrolyte consumption is inevitable for the formation of fresh SEI which culminates in the rapid failure of the cell.

An in situ optical micrograph test was carried out to observe the innate Li deposition behavior and further investigate the remarkable lithiophilicity of the CLCS. Figure 3g displays the initial state of the CLCS before the Li is plated on the skeleton. A homogeneous color change in the substrate was observed when metallic Li was plated on the CLCS for 30 min because of the favorable Li distribution and uniform Li deposition (Fig. 3h). As shown in Fig. 3i, bare CF exhibits a shiny color before the Li plating on the skeleton. However, the majority of the bare CF was not covered by metallic Li after continuous Li deposition for 30 min and was still exposed to the electrolyte (Fig. 3j), further corroborating its poor lithiophilicity compared to CLCS. Although the superior lithiophilicity of CLCS has been confirmed, the priority of Li deposition in this carbon-based material needs further examination with defects in the carbon playing an important role in storing Li ions. Hence, in situ Raman spectra were investigated as an appropriate probing method to determine the priority of the Li deposition behavior. Figure S11 displays the discharging and charging profiles of the CLCS at a current density of 1 mA cm^−2^ with an areal capacity of 3 mAh cm^−2^ in an assembled battery. As highlighted in Fig. 3k, the intensity of the D-band and G-band of the CLCS gradually decreased with the increase in Li plating time. Notably, the intensity of the D-band dropped faster than that of the G-band, indicating that metallic Li was preferentially deposited at the defects of CLCS [45]. Additionally, the intensity of the G-band was found to be enhanced before the D-band during the inverse charging process (Fig. 3l). The results of in situ Raman spectroscopy clearly demonstrate that metallic Li was first deposited on the defects, and gradually covered the surface of the CLCS. Hence, the stepwise Li deposition and stripping in the CLCS could avoid concentrated nucleation and the growth of metallic Li, effectively suppressing the generation of Li dendrites and the excess consumption of the electrolyte.

### Electrochemical Performances of CLCS-based Li Metal Batteries

**Fig. 4:**
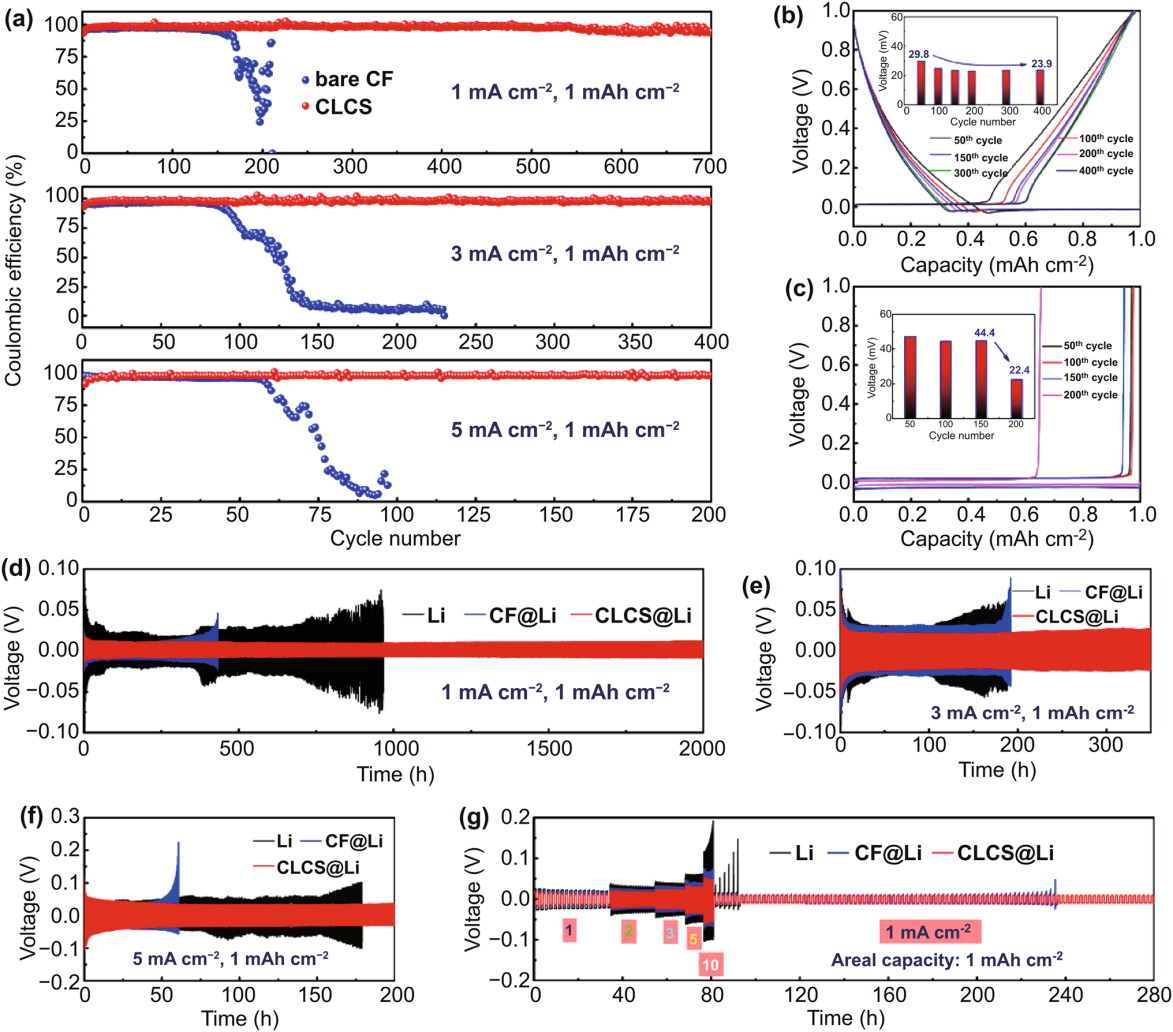
**a** Coulombic efficiency performance of bare CF and CLCS at 1, 3, and 5 mA cm^−2^ with a fixed capacity of 1 mAh cm^−2^. Voltage–capacity plots at different cycles and corresponding voltage hysteresis of **b** CLCS and **c** CF. Voltage–time profiles in symmetric cells composing of Li, CF@Li and CLCS@Li electrodes at **d** 1 mA cm^−2^, **e** 3 mA cm^−2^ and **f** 5 mA cm^−2^. **g** Rate performance with a cycling capacity of 1 mAh cm^−2^

The excellent lithiophilicity should enhance the electrochemical performance of the asymmetric and symmetric cells based on the CLCS. The reversibility of the Li plating/stripping performance on the substrate is an essential factor for evaluating the formation of Li dendrites or dead Li in the current collector of Li metal anodes. Figure S12 shows the Nyquist plots of asymmetric cells based on bare CF and CLCS electrodes. The CLCS electrode delivers a higher transfer impedance of 54.7 Ω than that (37.3 Ω) of CF due to the existence of nitrogen-doped carbon coating, which is less conductive than Cu. Moreover, Nyquist plot of CLCS electrodes after 50 cycles can be observed in Fig. S13. Because of the interface stabilization, transfer impedance has been decreased to 21.2 Ω from the initial state (54.7 Ω). This reduced transfer impedance is beneficial to promoting the uniform Li deposition. As shown in Fig. 4a, the asymmetric cells consisting of CLCS electrodes exhibit comparatively stable CE plots. Specifically, CLCS electrodes demonstrate a high CE value of 97.8% for 700 cycles at 1 mA cm^−2^, 97.5% for 400 cycles at 3 mA cm^−2^, and 98.1% for 200 cycles at 5 mA cm^−2^, respectively. Conversely, the bare CF electrode maintains a shorter cycling lifespan with a quick CE decay because of the generation of Li dendrites. The CLCS displays a superior CE performance to that of CF even when cycled at a high areal capacity of 3 mAh cm^−2^ (Fig. S14). Figure 4b displays the voltage profiles of the CLCS at different cycles at 1 mA cm^−2^ with a plating capacity of 1 mAh cm^−2^, which demonstrate that the CLCS maintains a stable and high CE as the cycle number increases. Additionally, the voltage polarization was found to decrease from 29.8 mV at the 50th cycle to 23.9 mV at the 400th cycle, which may result from the gradual stabilization of the electrode interface. Apart from the continuous capacity loss of stripped Li metal, the voltage profiles at different cycles of the bare CF are displayed in Fig. 4c and indicate a rapid voltage polarization drop from 44 mV at the 150th cycle to 22.4 mV at the 200th cycle, indicating an internal short circuit caused by dendritic Li. Symmetric cells based on CLCS@Li were assembled to determine the effect of improving the cycling stability and avoiding the generation of Li dendrites on the electrochemical performance. The CLCS@Li|CLCS@Li demonstrated an extremely stable voltage curve with an ultra-long cycling lifespan of 2000 h compared to the fluctuating voltage curves of Li|Li and CF@Li|CF@Li cells (Fig. 4d). Furthermore, even after 1000 cycles, a relatively small voltage hysteresis of 20.3 mV was maintained (Fig. S15). The CLCS@Li|CLCS@Li cells exhibited a long-term cycling performance with enhanced stability when cycled at a high current density of 3 mA cm^−2^ and 5 mA cm^−2^, similar to that at 1 mA cm^−2^ (Fig. 4e, f). However, the CF@Li|CF@Li cell experienced a rapid increase in voltage polarization after less than 200 h at 3 mA cm^−2^ and at 60 h at 5 mA cm^−2^ because of the uncontrolled growth of Li dendrites and excessive consumption of the electrolyte. The fragile SEI on the metallic Li foil was easily broken due to the large volume expansion of the plated Li metal, which diminishes the electrochemical performance of the Li|Li symmetric cell. Moreover, the CLCS@Li|CLCS@Li cell exhibits an impressive rate performance with slight fluctuations in voltage polarization. Notably, a symmetrical cell containing CLCS@Li exhibited a relatively low voltage hysteresis of approximately 115 mV at 10 mA cm^−2^ (Fig. 4g). These results indicate that CLCS with superior lithiophilicity can improve the electrochemical performance and extend the cycling life of working batteries.

**Fig. 5:**
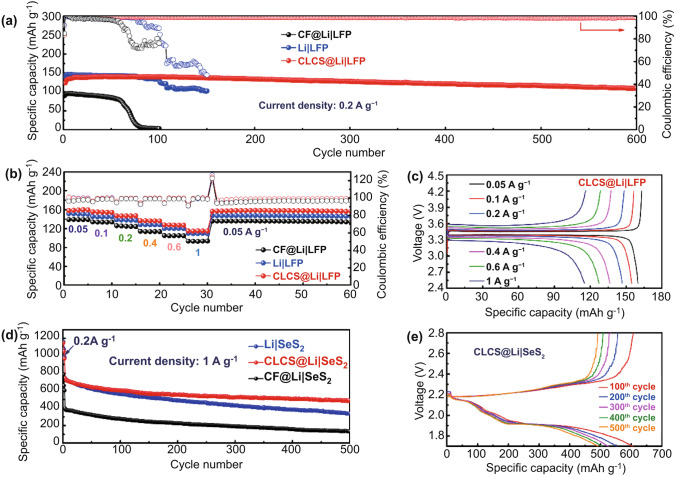
**a** Cycling performance of Li|LFP, CF@Li|LFP and CLCS@Li|LFP full cells at a current density of 0.2 A g^−1^. **b** Rate performance at various current densities from 0.05 to 1 A g^−1^ and **c** related galvanostatic charge–discharge profiles. **d** SeS_2_-based full cells coupled with Li, CF@Li and CLCS@Li anode at 1 A g^−1^. **e** Corresponding galvanostatic charge–discharge profiles at different cycles

A CLCS@Li anode was coupled to a commercial LiFePO_4_ (LFP) cathode and assembled into a full cell to determine its potential in practical Li metal batteries. For the CLCS@Li anode, metallic Li of 3 mAh cm^−2^ should be electroplated onto the skeleton. Additionally, the specific capacity of the CLCS@Li composite anode was measured to be around 1600 mAh g^−1^. The specific capacity of the unique anode is much superior to other types of anodes with Cu current collector. High density of Cu does have a negative effect on the specific capacity of composite anode. In order to better realize the practical application of high-energy Li metal batteries, it is essential to explore more light-weight current collectors. As displayed in Fig. 5a, CLCS@Li|LFP demonstrates a comparatively stable capacity plot with a high CE value at a current density of 0.2 A g^−1^. A high specific capacity of 110.1 mAh g^−1^ was obtained by the CLCS@Li|LFP full cell even after cycling 600 cycles. Additionally, a superior capacity retention ratio of 88.8% (compared to the initial specific capacity of 124 mAh g^−1^) indicates the suppressed formation of Li dendrites and dead Li, in contrast to the rapid capacity loss with sharp fluctuations for CF@Li|LFP and Li|LFP. Figure 5b displays the rate performance of full cells based on Li, CF@Li, and CLCS@Li anodes. The CLCS@Li|LFP cell displayed the highest specific capacity at various current densities compared to those of the Li|LFP and CF@Li|LFP full cells. The high capacity delivery and excellent capacity preservation exhibited by the CLCS@Li|LFP cell following the charge–discharge test at higher current density further demonstrate its improved electrochemical performance. Figure 5c displays the galvanostatic charge–discharge profiles at different current densities of CLCS@Li|LFP. The exceptional interfacial reaction kinetics increases the voltage polarization by approximately 30 mV from 0.05 to 1 A g^−1^. The CLCS@Li anode was also coupled with a selenium disulfide (SeS_2_) cathode to assemble a different full cell to gauge its applicability. As shown in Fig. 5d, the electrochemical performances of Li|SeS_2_, CF@Li|SeS_2_, and CLCS@Li|SeS_2_ cells were measured at a high current density of 1 A g^−1^. The CLCS@Li|SeS_2_ cell exhibited the slowest capacity decay and the highest capacity retention (66.3%) after 500 cycles among these full cells, while the initial cell activation was at 0.2 A g^−1^ for 3 cycles. A comparable Li|SeS_2_ cell has similar capacity decay during the first 50 cycles, benefiting from the initially stable electrode interface formed on the Li foil. The interface is then broken down by the growing Li dendrites owing to the uneven Li deposition, unavoidably leading to additional electrolyte consumption. The specific capacity of the Li|SeS_2_ cell decreases rapidly and continuously. This could be because the high amounts of dendritic Li originally on the anode, with the exception of the fragile CF@Li anode interface, are transferred onto the inactive dead Li during the continuous charge–discharge process, resulting in the extremely low capacity. Figure 5e exhibits the galvanostatic charge–discharge profiles at different cycles of the CLCS@Li|SeS_2_ cell. This cell demonstrates a high specific capacity (491.8 mAh g^−1^) even after cycling for 500 cycles with minor voltage polarization fluctuations between the 100th and the 500th cycle, in sharp contrast to the significant changes in the voltage polarization of the Li|SeS_2_ and CF@Li|SeS_2_ cells (Figs. S16 and S17). The electrochemical performances and thorough discussion on the cells employing CLCS-based Li anodes have also been proved the excellent lithiophilicity and effectively suppressed formation of Li dendrites, highlighting their potential as efficient and realistic Li metal batteries.

## Conclusion

In summary, we report the synthesis of a unique free-standing cucumber-like lithiophilic composite skeleton to function as a container for deposited Li metal through a facile fabrication method. The pyridinic N, pyrrolic N, and several Cu_x_N sites provide excellent lithiophilicity to facilitate strong binding to the Li atoms, guide uniform Li deposition and suppress the generation of Li dendrites. Furthermore, stepwise Li deposition and stripping on the CLCS, as determined by in situ Raman spectra, alleviate the concentrated and uncontrolled Li nucleation and growth. The cells consisting of CLCS electrodes benefit from these advantages and display prolonged cycling life and improved electrochemical performance. Additionally, they demonstrate an extremely high CE performance of 97.3% after 700 cycles in an asymmetrical cell along with an ultra-long and stable lifespan of 2000 h for a symmetrical cell at 1 mA cm^−2^ with a deposition capacity of 1 mAh cm^−2^. The full cells based on the CLCS@Li anodes assembled with LiFePO_4_ and SeS_2_ cathodes maintain a high reversible capacity of 110.1 mAh g^−1^ after 600 cycles in the CLCS@Li|LFP cell and 491.8 mAh g^−1^ after 500 cycles in the CLCS@Li|SeS_2_ cell. The successful construction and application of CLCS provide a template for dendrite-free and high-capacity Li metal batteries.

## Supplementary Information

Below is the link to the electronic supplementary material.

Supplementary file1 (PDF 1145 KB)
