# Supplementary material for: Dendrite-Free and Stable Lithium Metal Battery Achieved by a Model of Stepwise Lithium Deposition and Stripping
Source: Nanomicro Lett. 2021 Aug 9;13:170. doi: 10.1007/s40820-021-00687-3 (PMC8353026; doi:10.1007/s40820-021-00687-3)
Supplement: Supplementary file 1 — Supplementary file1 (PDF 1145 KB) [file 40820_2021_687_MOESM1_ESM.pdf]

Supporting Information for

Dendrite-Free and Stable Lithium Metal Battery Achieved by a Model of Stepwise Lithium Deposition and Stripping

Tiancun Liu[1], Jinlong Wang[1], Yi Xu[1], Yifan Zhang[1], Yong Wang[1, 2,] *

[1]Department of Chemical Engineering, School of Environmental and Chemical Engineering, Shanghai University, 99 Shangda Road, Shanghai 200444, People's Republic of China

[2]Key Laboratory of Organic Compound Pollution Control Engineering (MOE), 99 Shangda Road, Shanghai 200444, People's Republic of China

*Corresponding author. E-mail: yongwang@shu.edu.cn (Yong Wang)

Supplementary Figures

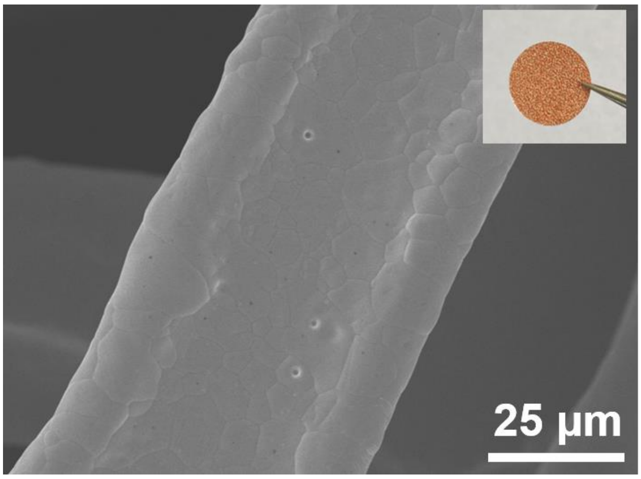

Fig. S1 SEM image of bare CF and related optical image (insertion part)

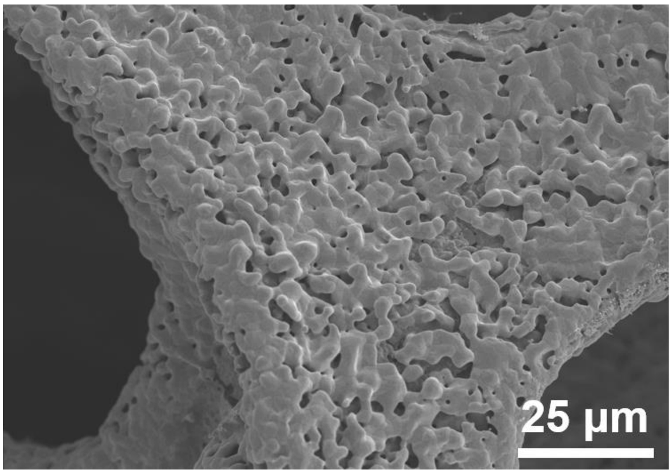

Fig. S2 SEM image of reduced CF after being calcinated in the mixed gas of H_2 and Ar (5%/95%, v/v) at 600 °C for 2 h

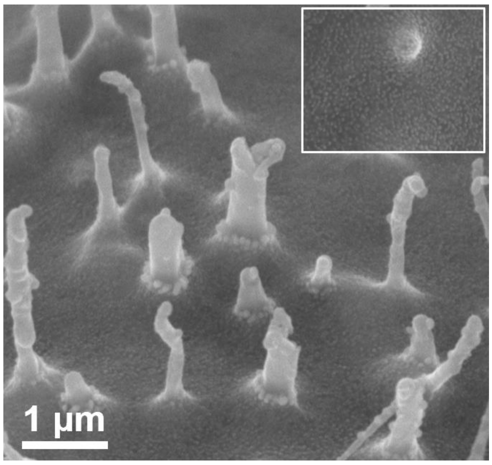

Fig. S3 SEM image of CLCS at a higher magnification, in which the surface of CLCS is covered by massive N-doped carbon coated Cu nanoparticles

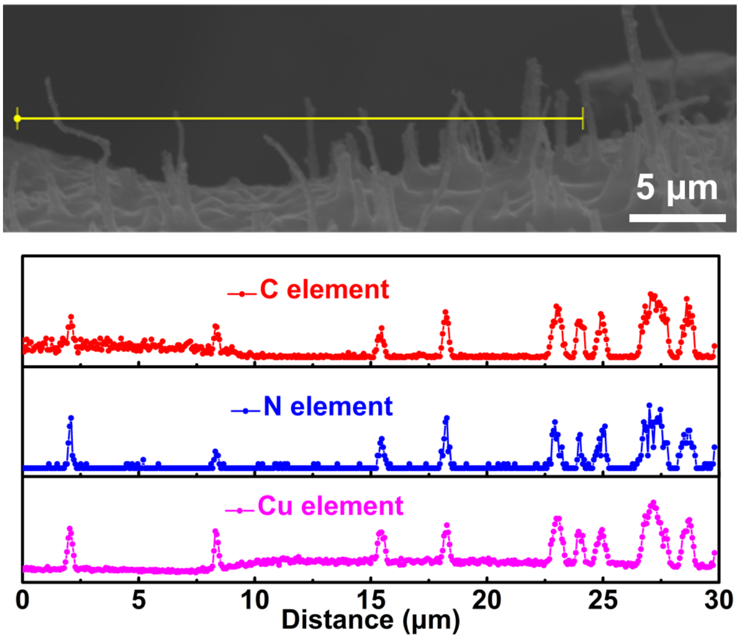

Fig. S4 SEM image of the selected area in CLCS and the corresponding line-scanning spectrum of C, N and Cu elements

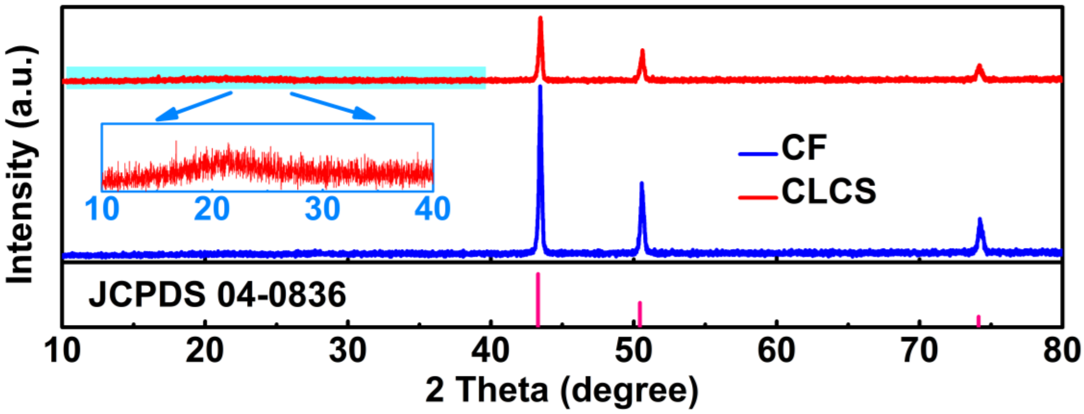

Fig. S5 XRD patterns of bare CF and CLCS

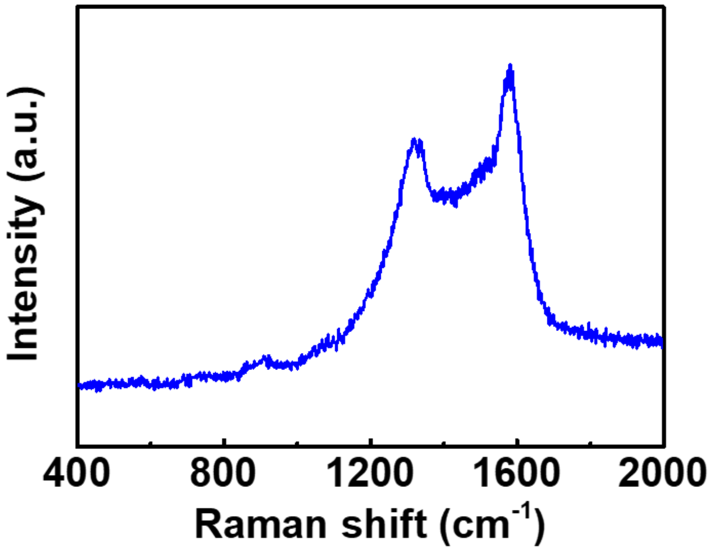

Fig. S6 Raman spectrum of CLCS

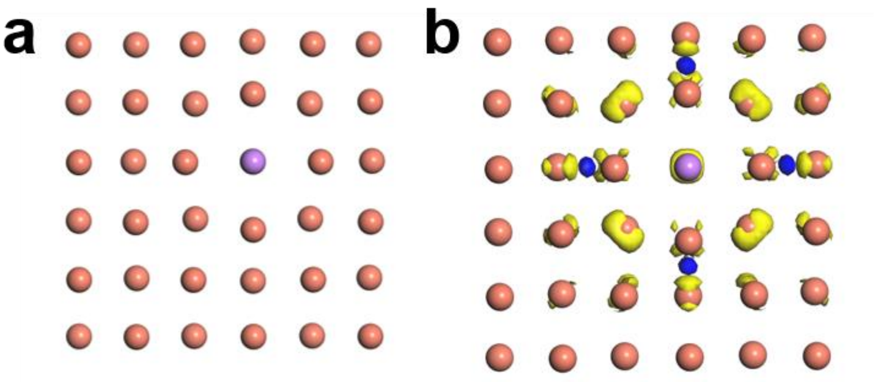

Fig. S7 Density functional theory calculations on the adsorption energy between Li atoms and Cu metal, in which the adsorption energy is calculate at 2.597 eV

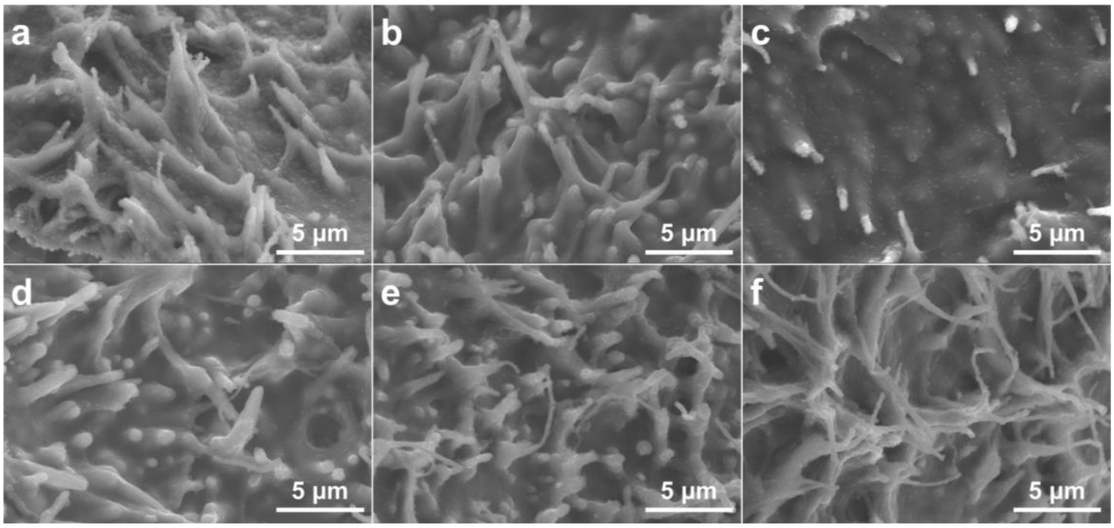

Fig. S8 SEM images of CLCS plating with Li deposition of **a** 1 mAh cm^{-2}, **b** 2 mAh cm^{-2} and **c** 3 mAh cm^{-2}. SEM images of CLCS during stripping with residual metallic Li of **d** 2 mAh cm^{-2}, **e** 1.5 mAh cm^{-2} and **f** 1 mAh cm^{-2}

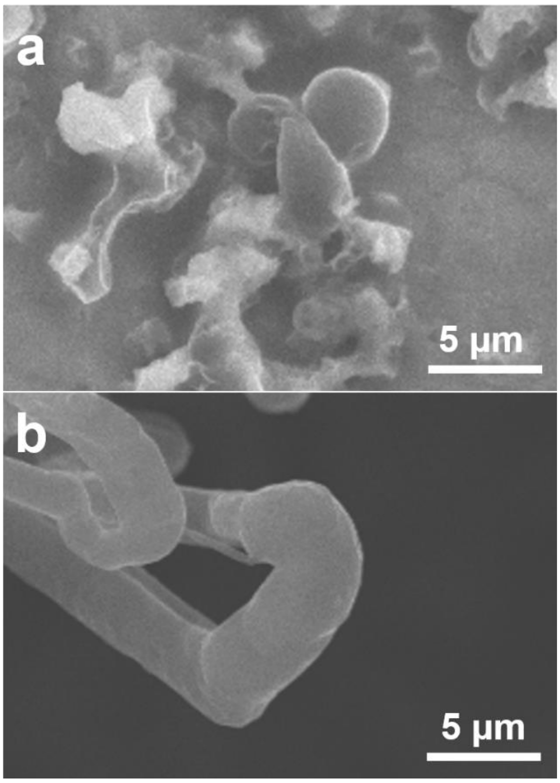

Fig. S9 SEM images showing the enlarged detail of bare CF loading with Li deposition of **a** 1 mAh cm^{-2} and **b** 2 mAh cm^{-2}

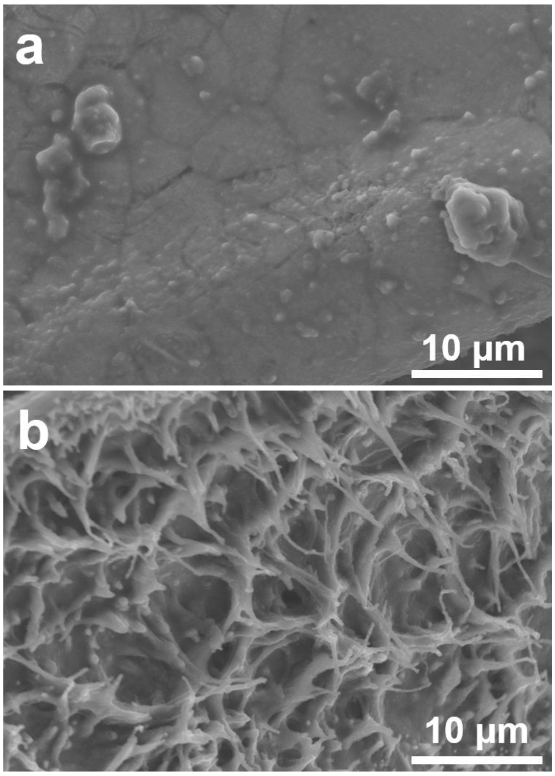

Fig. S10 SEM images exhibit the morphology of **a** the cycled CF and **b** CLCS after 50 cycles depicting prominent residual Li on the skeleton of the CF in contrast to the residual-free CLCS

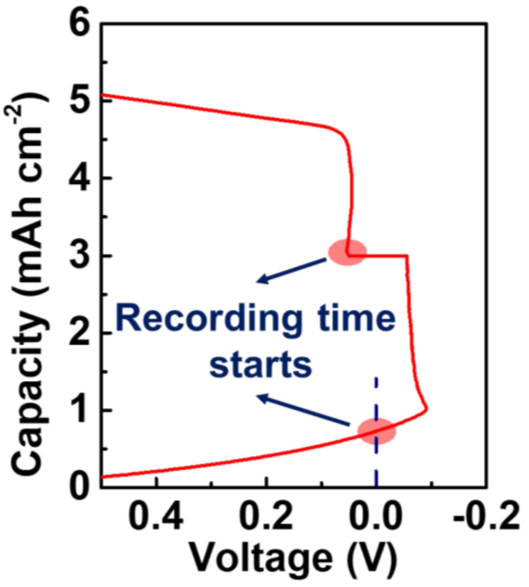

Fig. S11 Capacity-voltage profile of CLCS in asymmetric cell at the current density of 1 mA cm^{-2}

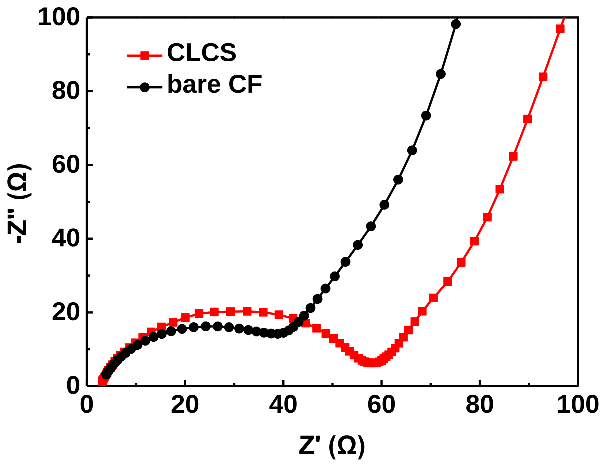

Fig. S12 Nyquist plots of CLCS and bare CF electrodes

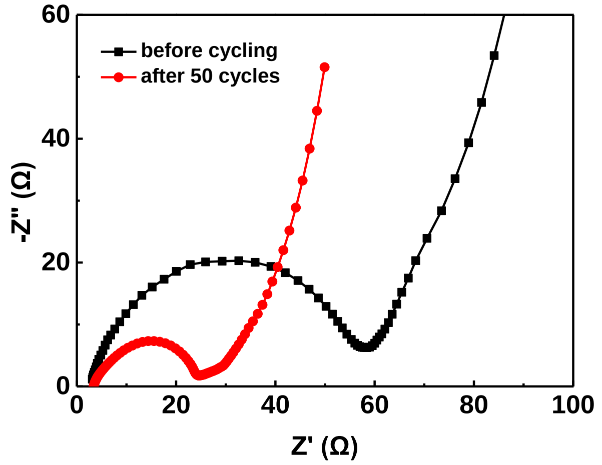

Fig. S13 Nyquist plots of CLCS before cycling and after 50 cycles

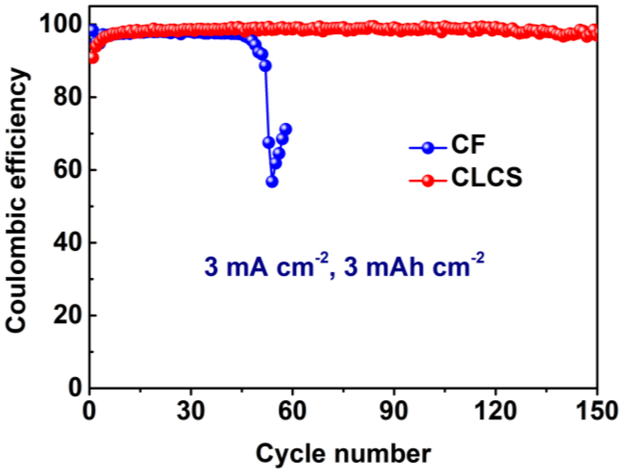

Fig. S14 CE performance of CLCS and bare CF at 3 mA cm^{-2} with a cycling capacity of 3 mAh cm^{-2}. There is an ultra-stable cycling plot with high CE value in the asymmetric cell of CLCS|Li

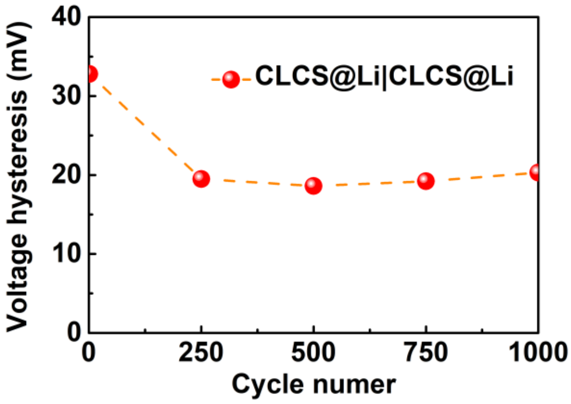

Fig. S15 Voltage hysteresis of symmetric cell composing of CLCS@Li electrodes, in which quite small value of 20.3 mV can be maintained even after 1000 cycles

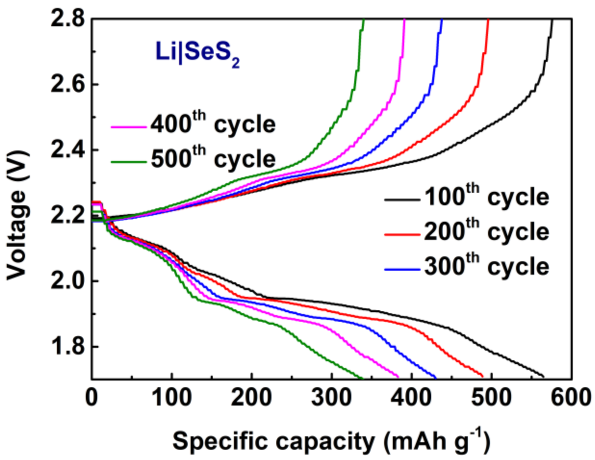

Fig. S16 Voltage-capacity plots at different cycles of CF@Li|SeS_2 full cell

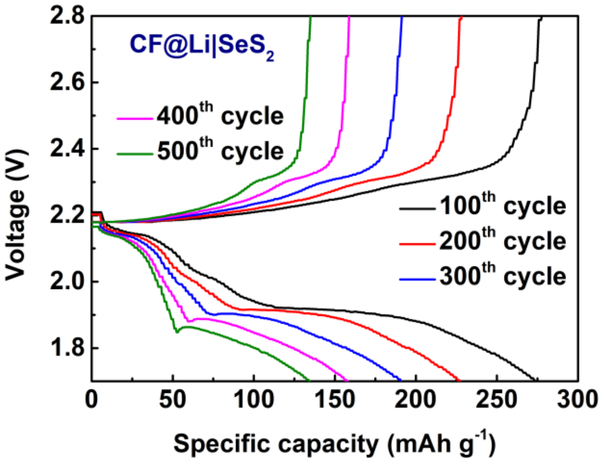

Fig. S17 Voltage-capacity plots at different cycles of Li|SeS_2 full cell
